# Difference in mortality risk predicted by leukocyte and lymphocyte levels in COVID-19 patients infected with the Wild-type, Delta, and Omicron strains

**DOI:** 10.1097/MD.0000000000037516

**Published:** 2024-03-08

**Authors:** Hongjun Zhang, Yanjun Zhao, Wenjie Li, Yaqin Chai, Xing Gu

**Affiliations:** a Respiratory and Critical Care Medicine, Xi’an Chest Hospital, Xi’an, Shaanxi, PR China; b Infectious Disease Department, Wuhan Huoshenshan Hospital, Wuhan, Hubei, PR China.

**Keywords:** COVID-19, leukocyte, lymphocyte, predict, SARS-CoV-2

## Abstract

This study aimed to investigate the changing trends, level differences, and prognostic performance of the leukocyte and lymphocyte levels of patients infected with the Wild strains, Delta strains and Omicron strains to provide a reference for prognostic assessment. In the current study, we conducted a retrospective cross-sectional study to evaluate the changing trends, level differences, and prognostic performance of leukocyte and lymphocyte of different strains at admission and discharge may already exist in patients with coronavirus disease-2019 (COVID-19) infected with the Wild type, Delta, and Omicron strains. A retrospective cross-sectional study was conducted. We recruited and screened the 243 cases infected with the Wild-type strains in Wuhan, the 629 cases infected with the Delta and 116 cases infected strains with the Omicron strains in Xi’an. The leukocyte and lymphocyte levels were compared the cohort of Wild-type infection with the cohort of Delta and the Omicron. The changes in the levels of leukocytes and lymphocytes exhibit a completely opposite trend in patients with COVID-19 infected with the different strains. The lymphocyte level at admission and discharge in patients with COVID-19 infected with Omicron strains (area under curve [AUC] receiver operating characteristic curve [ROC] 72.8–90.2%, 82.8–97.2%) presented better performance compared patients with COVID-19 infected with Wild type strains (AUC ROC 60.9–80.7%, 82.3–97.2%) and Delta strains (AUC ROC 56.1–84.7%, 40.3–93.3%). Kaplan–Meier curves showed that the leukocyte levels above newly established cutoff values and the lymphocyte levels below newly established cutoff values had a significantly higher risk of in-hospital mortality in COVID-19 patients with Wild-type and Omicron strains (*P* < .01). The levels of leukocyte and lymphocyte at admission and discharge in patients with COVID-19 infected with the Wild type, Delta, and Omicron strains may be differences among strains, which indicates different death risks. Our research may help clinicians identify patients with a poor prognosis for severe acute respiratory syndrome coronavirus 2 infection.

## 1. Introduction

Since the onset of the wild-type strain of severe acute respiratory syndrome coronavirus 2 (SARS-CoV-2) in December 2019, which causes coronavirus disease 2019 (COVID-19), there have been several globally circulating multiple new variants of concern (VOC). To date, there have been 5 main lineages that are identified as the VOC by the World Health Organization, such as B.1.1.7 (Alpha),^[1]^ B.1.351 (Beta),^[2]^ P.1 (Gamma),^[3]^ B.1.617.2 (Delta),^[4]^ and B..1.1.529 (Omicron).^[5]^ The variants are associated with increased transmissibility.^[6]^ The Delta first reported in India in December 2020 and was fast becoming the dominant strain in many countries and regions.^[7]^ And the Omicron variant of SARS-CoV-2 was first identified in South Africa and Botswana and was reported to the World Health Organization on November 24, 2021, as a novel variant.^[8,9]^ Owing to its enhanced transmissibility, Omicron has rapidly replaced Delta as the dominant variant on a global scale.^[10]^

With vaccine and specific drugs roll-out, antibody tests (which detect the host’s response to infection or vaccination) has become a useful monitoring tool to inform public policy, all these measures have made the COVID-19 epidemic gradually from pandemic response to pandemic control.^[11]^ Although the epidemic prevention and control policies of countries and regions worldwide have corresponding changed, the continuous evolution and mutation of the SARS-CoV-2, as well as the resulting variant strains with their enhanced transmission, pathogenicity and immune escape have still posed a serious threat to human health.^[6,12,13]^ Through in-depth research, we have gained more knowledge and understanding of the epidemic situation, transmission characteristics and clinical features of the SARS-CoV-2 wild type, Delta, and Omicron strains. However, there is a lack of detailed report on the characteristics of leukocytes and lymphocytes of patients infected with them living in Xi’an City and Wuhan City, China. To provides a reference for the prevention and treatment of COVID-19 in this region, leukocyte and lymphocyte counts of COVID-19 patients living in Xi’an City and Wuhan City, were obtained in the present study.

In December 2021 and December 2022, Delta and Omicron each triggered a new wave of outbreak in Xi’an, Shaanxi Province. The local outbreak in Xi’an, mainly transferred to Xi’an chest hospital, the official designated hospital to manage the SARS-CoV-2 patients, comprised 745 locally transmitted cases (629 cases infected with the Delta strain and 116 cases of Omicron strain) during the study period. All patients were confirmed infected with the same strain of the Delta and Omicron by next-generation sequencing. In addition, I also was lucky enough to go to the Wuhan Huoshenshan Hospital, only one of official designated hospitals, to treat COVID-19 with the wild-type strain in 2020. Therefore, our study provided an incredible opportunity to explore their difference in leukocyte and lymphocyte counts among patients infected with Wild-type, Delta, and Omicron strains.

## 2. Materials and methods

### 2.1. Patients’ involvement and demographics characteristics

All hospitalized patients (n = 988), diagnosed with COVID-19 based on regions with prevalent epidemic and positive nucleic acid detection results were involved in this study. Among them, 243 cases (admission date from February 10 to March 8, 2020) were from Huoshenshan Hospital of Wuhan, Huoshenshan Hospital of Wuhan was a new hospital for hospitalizing patients with COVID-19 after completion on February 2, 2020, and 629 cases (admission date From December 22, 2021, to February 17, 2022) 116 cases (admission date From December 28, 2022, to January 29, 2023) were from in Xi’an Chest Hospital. All patients involved in this study were living in Wuhan and Xi’an during the outbreak period of COVID-19. Patients in Xi’an were the local residents and epidemiologically confirmed to be linked to delta strain and omicron strain. Meanwhile, a Wild-type strain cohort from the Huoshenshan hospital two years ago was also included for comparison. The Wild-type strain cohort consisted of all the cases with complete medical records from February to March 2020, the first wave of the pandemic in China.

These data show that 24.6% cases were Wild-type strain, 63.7% were Delta strain and 11.7% were Omicron strain. Among the patients infected with the Wild-type, Delta, and Omicron strain, non-survivors and survivors were 18.1% and 81.9%, 98.6% and 1.4%, 68.1% and 31.9%, respectively. The age distribution characteristics of the 3 waves of COVID-19 in patients over and under 60 years old were 21.8% and 78.2%, 87.6% and 12.4%, 39.7% and 60.9%, respectively. And the gender distribution of the 3 waves of COVID-19 in male and female patients was 56.0% and 44.0%, 54.5% and 45.5%, 69.0%, and 31.0% respectively.

All patients were confirmed by the local Centers for Disease Control and transferred to Huoshenshan hospital and Xi’an Chest Hospital, the official designated hospital to manage the COVID-19 in Wuhan and Xi’an. Referring to guidelines issued by Chinese National Health Commission (Trial Version 7&8). Severe COVID-19 was designated when the patients had one of the following criteria: (a) respiratory distress with respiratory frequency ≥ 30/min; (b) pulse oximeter oxygen saturation ≤ 93% at rest; and (c) oxygenation index (artery partial pressure of oxygen/inspired oxygen fraction) ≤ 300 mm Hg. Critical COVID-19 was designated when the patients had one of the following criteria: (a) respiratory failure with mechanical ventilation; (b) shock; and (c) combination with other organ failure; requirement of Intensive Care Unit for monitoring and treatment.

### 2.2. Laboratory testing

Blood testing for all patients was performed by the clinical laboratory of Huoshenshan Hospital of Wuhan and Xi’an Chest Hospital. Medical laboratory results in this study, including the levels of leukocytes and lymphocytes, were collected for each non-survivors and survivors. Laboratory findings were derived from the electronic medical charts. The data were extracted by 2 independent clinicians into the electronic database. Any major dispute was resolved by consultation with a third reviewer.

### 2.3. Ethical approval

The study was approved by the Ethics Committee for Scientific Research at Xi’an Chest Hospital (No. S2022-0010).

Clinical and laboratory data collection was approved by Huoshenshan Hospital, Wuhan. Moreover, patient names, identification numbers, work units, home addresses, contact persons, and telephone information were anonymized. In addition, Huoshenshan Hospital of Wuhan has stopped running before the article is completed. Therefore, written informed consent was not required in accordance with local guidelines.

### 2.4. Statistical analysis

Data were analyzed in SPSS 23.0 (SPSS Inc., Chicago). To compare continuous variable data in different patient groups, variance analyses and Kruskal–Wallis tests were performed.

To better understand the overall performance of leukocytes and lymphocytes, we performed standard receiver-operating characteristic (ROC) curve analyses in R software (Version 4.2.2).^[14,15]^ The area under the ROC curve (AUC) was calculated to evaluate leukocytes and lymphocytes performance. The best cutoff points were evaluated using standard ROC analyses and determined using Youden index maximization. The AUC curves with 95% confidence intervals (CI) were used to compare the prognostic accuracy of leukocytes and lymphocytes.

Kaplan–Meier curves and log rank tests were generated in GraphPad Prism software (Version 5.0) to evaluate cumulative survival curves related to in-patient deaths. Risk factor risk ratios (HR) were calculated using univariate Cox proportional risk regression analyses and mixed effect Cox models in R software (version 4.2.2).

Scatter diagrams and survival analyses were processed in GraphPad Prism software (version 5.0, GraphPad Software Inc). *P* < .05 values were considered statistically significant.

## 3. Results

### 3.1. The admission- and discharge-stage levels of leukocyte and lymphocyte in survivors and non-survivors infected with the Wild-type, Delta, and Omicron strain

**Figure 1.**
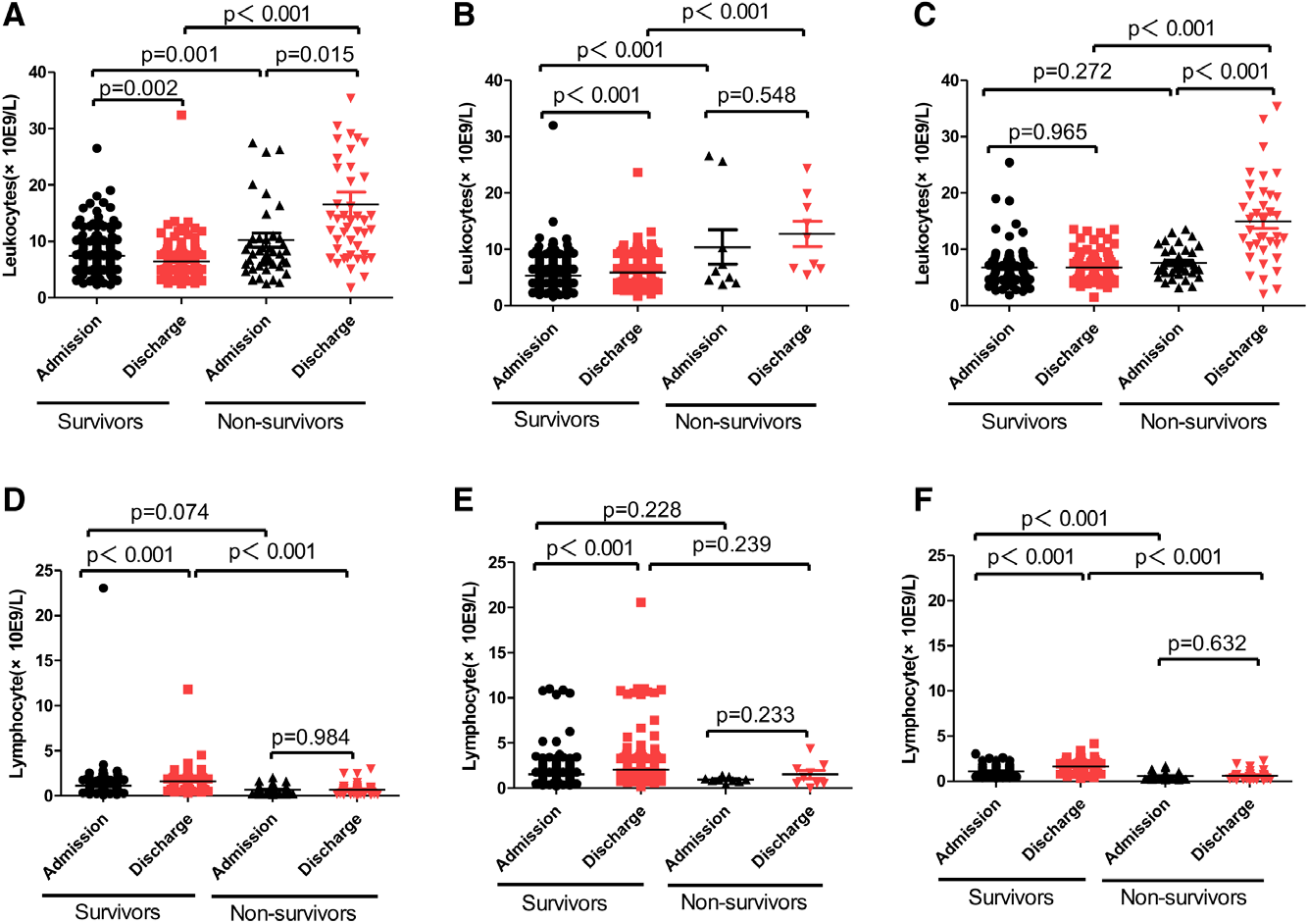
Summary of leukocyte and lymphocyte levels in patients with COVID-19. (A) The admission- and discharge-stage levels of leukocyte in survivors and non-survivors infected with the Wild-type strain. (B) The admission- and discharge-stage levels of leukocyte in survivors and non-survivors infected with the Delta strain. (C) The admission- and discharge-stage levels of leukocyte in survivors and non-survivors infected with the Omicron strain. (D) The admission- and discharge-stage levels of lymphocyte in survivors and non-survivors infected with the Wild-type strain. (E) The admission- and discharge-stage levels of lymphocyte in survivors and non-survivors infected with the Delta strain. (F) The admission- and discharge-stage levels of lymphocyte in survivors and non-survivors infected with the Omicron strain.

The blood test results were shown in Figure 1. The leukocyte level of survivors at discharge was the same or lower than that at admission, while the leukocyte level of non-survivors at discharge was significantly higher than that at admission, in patients with COVID-19 infected with different strains (Fig. 1A, B, and C). On the contrary, in the patients with COVID-19 infected with different strains, the lymphocyte level of survivors at discharge was significantly higher than that at admission (*P* < .001), while the lymphocyte level of non-survivors at discharge was always at a low level than that at admission (*P* > .05, Fig. 1D, E, and F).

### 3.2. Differences in leukocyte and lymphocyte levels among patients infected with Wild-type, Delta, and Omicron strains at admission and discharge

**Figure 2.**
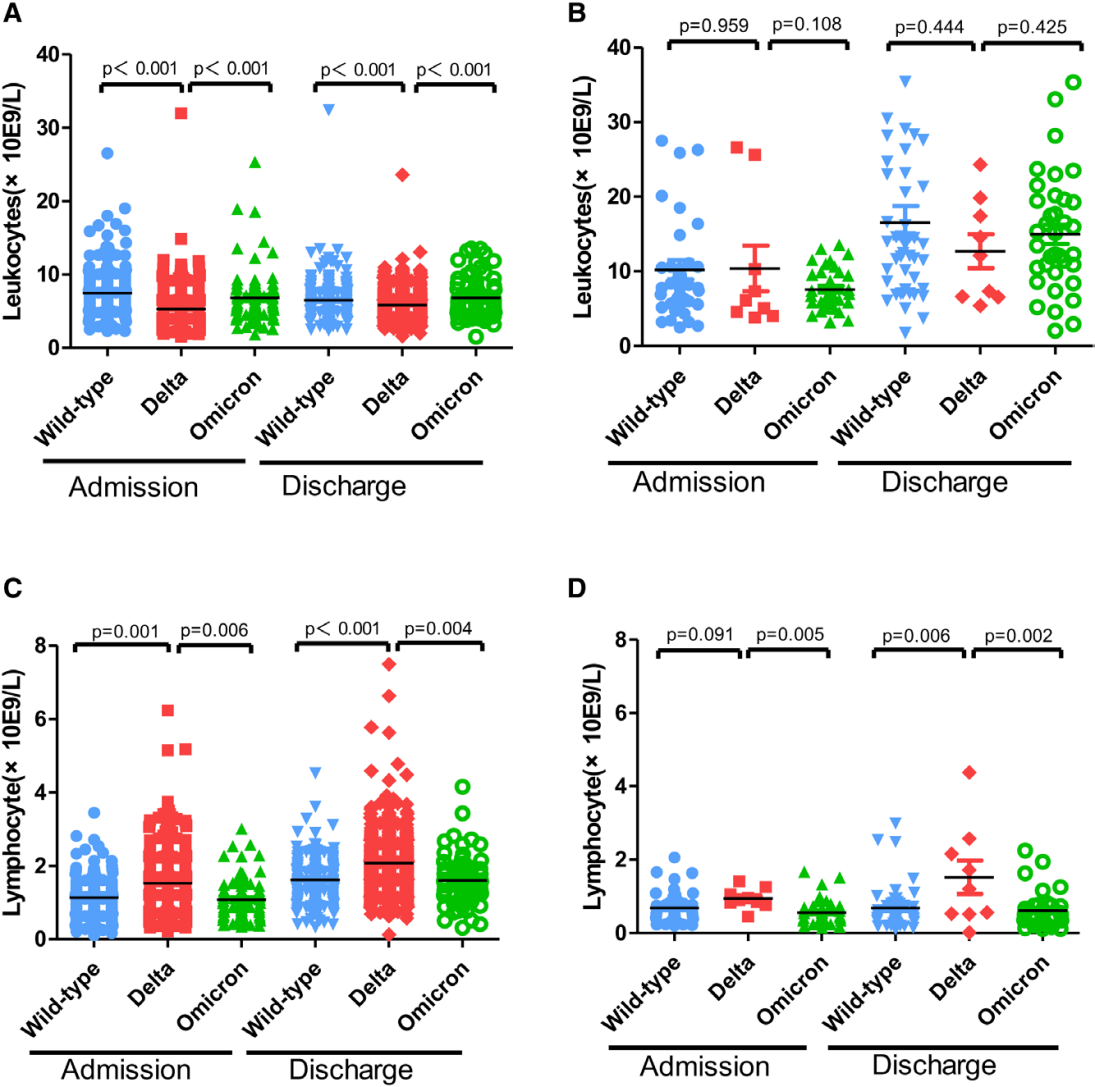
Summary of leukocyte and lymphocyte levels in COVID-19 patients infected with different strains. (A) The admission- and discharge-stage levels of leukocyte in survivors infected with the Wild-type, Delta, and Omicron strain. (B) The admission- and discharge-stage levels of leukocyte in non-survivors infected with the Wild-type, Delta, and Omicron strain. (C) The admission- and discharge-stage levels of lymphocyte in survivors infected with the Wild-type, Delta, and Omicron strain. (D) The admission- and discharge-stage levels of lymphocyte in non-survivors infected with the Wild-type, Delta, and Omicron strain.

In survivors with COVID-19, the admission- and discharge-stage levels of leukocyte of patients infected with the Delta strain was lower than that of patients infected with the Wild-type strain and Omicron strain (*P* < .001, Fig. 2A), while the admission- and discharge-stage levels of lymphocyte of patients infected with the Delta strain was higher than that of patients infected with the Wild-type strain and Omicron strain (*P* < .05, Fig. 2C). Additionally, in non-survivors with COVID-19, there was no difference in the admission- and discharge-stage levels of leukocyte of patients infected with the Wild-type, Delta, and Omicron strain (*P* > .05, Fig. 2B), while the admission- and discharge-stage levels of lymphocyte of patients infected with the Delta strain was higher than that of patients infected with the Omicron strain (*P* < .05, Fig. 2D). Compared to non-survivors infected with the Wild-type strain, there was no difference in the admission-stage levels of lymphocyte of patients with the Delta strain (*P* > .05, Fig. 2D), while the discharge-stage levels of lymphocyte of patients infected with the Delta strain was higher than that of patients infected with the Wild-type strain (P < .05, Fig. 2D).

### 3.3. Prognostic performance of leukocyte and lymphocyte in predicting in-hospital COVID-19 mortality in the patients with COVID-19 infected with different strains

**Figure 3.**
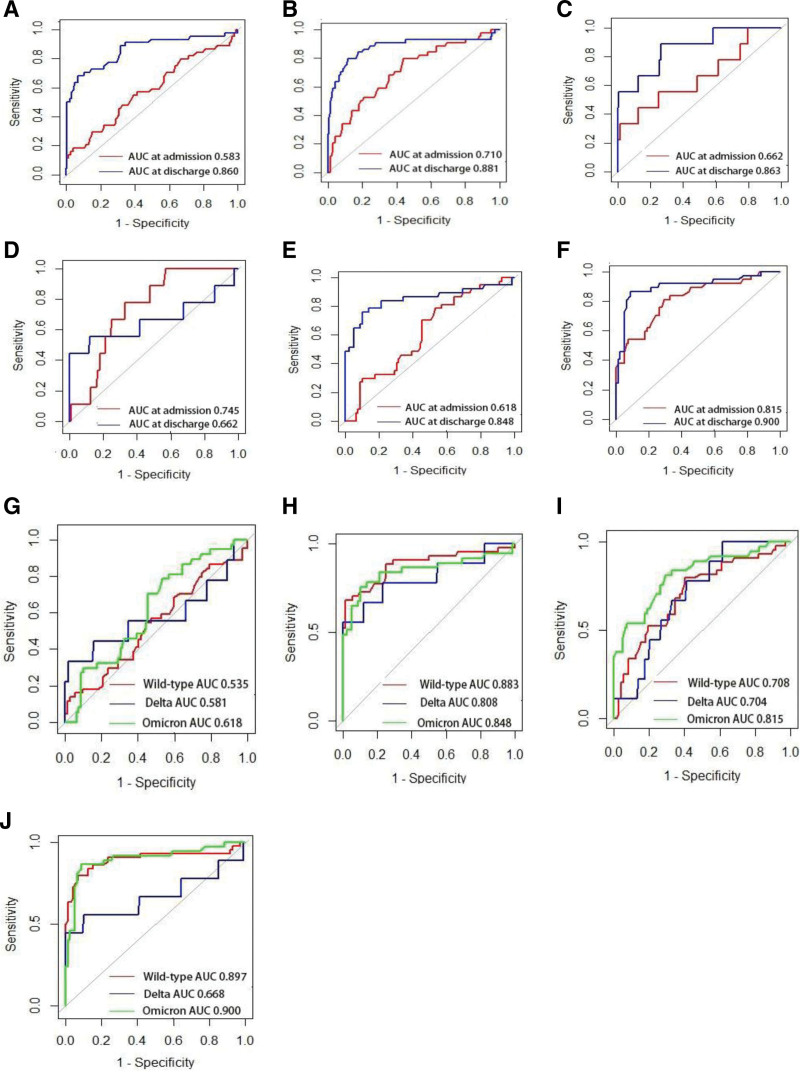
Standard ROC curve analysis based on the admission and discharge levels of leukocyte and lymphocyte in patients with Wild-type, Delta, and Omicron strain, respectively. (A and B) Standard ROC curve analysis based on the admission and discharge levels of leukocyte and lymphocyte in patients with Wild-type strain, respectively. (C and D) Standard ROC curve analysis based on the admission and discharge levels of leukocyte and lymphocyte in patients with Delta strain, respectively. (E and F) Standard ROC curve analysis based on the admission and discharge levels of leukocyte and lymphocyte in patients with Omicron strain, respectively. (G) Standard ROC curve analysis based on the admission levels of leukocyte in patients with Wild-type, Delta, and Omicron Strain. (H) Standard ROC curve analysis based on the discharge levels of leukocyte in patients with Wild-type, Delta, and Omicron Strain. (I) Standard ROC curve analysis based on the admission levels of lymphocyte in patients with Wild-type, Delta, and Omicron Strain. (J) Standard ROC curve analysis based on the discharge levels of lymphocyte in patients with Wild-type, Delta, and Omicron Strain. AUC = area under curve; ROC = receiver operating characteristic curve.

Standard ROC curve analyses were used, in survivors and non-survivors infected with the Wild-type, Delta, and Omicron strain, to calculate sensitivity, specificity, positive predictive values, and negative predictive values, diagnostic likelihood ratio, false positive, false negative, true positive, and true negative values of leukocyte and lymphocyte at the admission- and discharge-stage (Tables 1 and 2). And optimal cutoff values were calculated based on ROC curves (Tables 1 and 2). Overall leukocyte and lymphocyte performance is shown in Figure 3 and Tables 1 and 2.

**Table 1:** Overall performance of the leukocyte according to the standard ROC analysis.

	Wlid-type		Delta		Omicron	
	Admission	Discharge	Admission	Discharge	Admission	Discharge
N	243	243	699	699	116	116
Mortality	44	44	9	9	37	37
AUC (95% CI)	0.583 (0.483–0.683)	0.860 (0.787–0.934)	0.662 (0.447–0.877)	0.863 (0.731–0.994)	0.618 (0.512–0.725)	0.848 (0.899–0.757)
Cutoff (×10E9/L)	7.35	10.05	10.165	6.595	5.83	10.445
Sensitivity (%)	56.8	68.2	33.3	88.9	78.4	75.7
Specificity (%)	58.8	93.5	98.7	73.6	46.8	89.9
PPV (%)	22.6	68.3	25	0.5	40.8	77.8
NPV (%)	48.1	92.1	99.1	98.4	82.2	88.8
DLR.Positive	1.324	9.741	25.556	0.421	1.478	7.473
DLR.Negative	0.773	0.389	0.675	1.207	0.462	0.271
FP	82	13	9	182	78	8
FN	20	16	6	8	8	9
TP	24	28	3	1	29	28
TN	117	186	681	508	37	71

AUC = area under the ROC curves, CI = confidence interval, DLR = diagnostic likelihood ratio, FN = false negative, FP = false positive, N = number, NPV = negative predictive value, PPV = positive predictive value, TN = true negative, TP = true positive.

**Table 2:** Overall performance of the lymphocyte according to the standard ROC analysis.

	Wild-type		Delta		Omicron	
	Admission	Discharge	Admission	Discharge	Admission	Discharge
N	243	243	699	699	116	116
Mortality	44	44	9	9	37	37
AUC (95% CI)	0.710 (0.623–0.796)	0.881 (0.806–0.955)	0.745 (0.628–0.861)	0.662 (0.399–0.926)	0.815 (0.728–0.902)	0.900 (0.828–0.972)
Cutoff (×10E9/L)	0.87	0.86	1.055	0.57	0.74	0.845
Sensitivity (%)	79.5	79.5	77.8	44.4	81.8	86.5
Specificity (%)	56.3	88.4	67.1	99.9	70.9	91.1
PPV (%)	7.4	4.9	0.4	0.7	11.1	6.5
NPV (%)	71.3	39.7	97	0.2	43.4	17.9
DLR.Positive	0.363	0.231	0.331	0.556	0.267	3.624
DLR.Negative	1.819	6.882	2.364	306.7	2.785	9.761
FP	112	176	463	689	56	72
FN	35	35	7	4	30	32
TP	9	9	2	5	7	5
TN	87	23	227	1	23	7

AUC = area under the ROC curves, CI = confidence interval, DLR = diagnostic likelihood ratio, FN = false negative, FP = false positive, N = number, NPV = negative predictive value, PPV = positive predictive value, TN = true negative, TP = true positive.

1. Leukocyte and lymphocyte levels at the admission- and discharge-stage in patients infected with the Wild-type strain (Fig. 3A and D).

When incorporating leukocyte levels at the admission- and discharge-stage in survivors and non-survivors infected with the Wild-type strain, the AUC was 0.583 (95% CI: 0.483–0.683) and 0.860 (95% CI: 0.787–0.934), and optimal cutoffs were 7.35 × 10E9/L (sensitivity = 56.8% and specificity = 58.8%) and 10.05 × 10E9/L (sensitivity = 68.2% and specificity = 93.5%). When incorporating lymphocyte levels at the admission- and discharge-stage in survivors and non-survivors infected with the Wild-type strain, the AUC was 0.710 (95% CI: 0.623–0.796) and 0.881 (95% CI: 0.806–0.955), and optimal cutoffs were 0.87 × 10E9/L(sensitivity = 79.5% and specificity = 56.3%) and 0.86 × 10E9/L (sensitivity = 79.5% and specificity = 88.4%).

1. Leukocyte and lymphocyte levels at the admission- and discharge-stage in patients infected with the Delta strain (Fig. 3C and D).

When incorporating leukocyte levels at the admission- and discharge-stage in survivors and non-survivors infected with the Delta strain, the AUC was 0.662 (95% CI: 0.447–0.877) and 0.863 (95% CI: 0.731–0.994), and optimal cutoffs were 10.165 × 10E9/L(sensitivity = 33.3% and specificity = 98.7%) and 6.595 × 10E9/L (sensitivity = 88.9% and specificity = 73.6%). When incorporating lymphocyte levels at the admission- and discharge-stage in survivors and non-survivors infected with the Delta strain, the AUC was 0.745 (95% CI: 0.628–0.861) and 0.662 (95% CI: 0.399–0.962), and optimal cutoffs were 1.055 × 10E9/L(sensitivity = 77.8% and specificity = 67.1%) and 0.57 × 10E9/L (sensitivity = 44.4% and specificity = 99.9%).

1. Leukocyte and lymphocyte levels at the admission- and discharge-stage in patients infected with the Omicron strain (Fig. 3E and F).

When incorporating leukocyte levels at the admission- and discharge-stage in survivors and non-survivors infected with the Omicron strain, the AUC was 0.618 (95% CI: 0.512–0.725) and 0.848 (95% CI: 0.899–0.757), and optimal cutoffs were 5.83 × 10E9/L (sensitivity = 78.4% and specificity = 46.8%) and 10.445 × 10E9/L (sensitivity = 75.7% and specificity = 89.9%). When incorporating lymphocyte levels at the admission- and discharge-stage in survivors and non-survivors infected with the Omicron strain, the AUC was 0.815 (95% CI: 0.728–0.902) and 0.900 (95% CI: 0.828–0.972), and optimal cutoffs were 0.74 × 10E9/L(sensitivity = 81.8% and specificity = 70.9%) and 0.845 × 10E9/L (sensitivity = 86.5% and specificity = 91.1%).

### 3.4. Comparison of leukocyte and lymphocyte levels at the admission- and discharge-stage in patients infected with the Wild-type, Delta, and Omicron strain

Standard ROC curve analyses were used, in survivors and non-survivors infected with the Wild-type, Delta, and Omicron strain, to calculate AUC and 95% CI of leukocyte and lymphocyte at the admission- and discharge-stage. Their leukocyte and lymphocyte performance were shown in Figure 3G and H.

**Figure 4.**
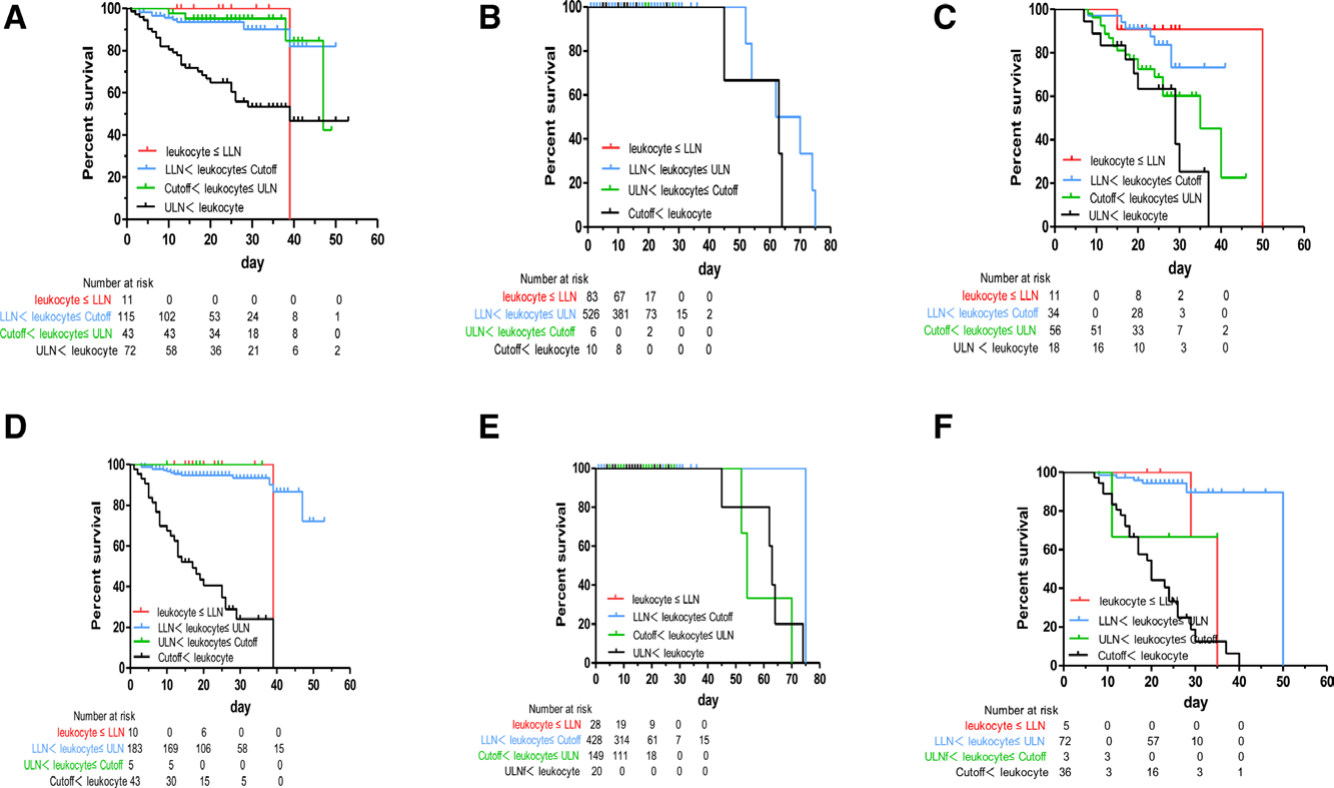
Kaplan–Meier curves showing the cumulative survival rates of patient groups divided by cutoff, LLN, and ULN. (A) Kaplan–Meier curve analysis based on the admission levels of leukocyte in patients with Wild-type strain. (B) Kaplan–Meier curve analysis based on the admission levels of leukocyte in patients with Delta strain. (C) Kaplan–Meier curve analysis based on the admission levels of leukocyte in patients with Omicron strain. (D) Kaplan–Meier curve analysis based on the discharge levels of leukocyte in patients with Wild-type strain. (E) Kaplan–Meier curve analysis based on the discharge levels of leukocyte in patients with Delta strain. (F) Kaplan–Meier curve analysis based on the discharge levels of leukocyte in patients with Omicron strain. LLN, low limit of normal; ULN, upper limit of normal.

For leukocyte at admission, the AUC was 0.535 (95% CI: 0.424–0.646) for the Wild-type strain, 0.581 (95% CI: 0.320–0.842) for the Delta strain, and 0.618 (95% CI: 0.512–0.725) for the Omicron strain (Fig. 3I). And, for leukocyte at discharge, the AUC was 0.883 (95% CI: 0.810–0.956) for the Wild-type strain, 0.808 (95% CI: 0.611–1.000) for the Delta strain, and 0.848 (95% CI: 0.755–0.942) for the Omicron strain (Fig. 4B). Similarly, the AUC was 0.708 (95% CI: 0.609–0.807) for the Wild-type strain, 0.704 (95% CI: 0.561–0.847) for the Delta strain, and 0.815 (95% CI: 0.728–0.902) for the Omicron strain for lymphocyte at admission (Fig. 4C). And, the AUC was 0.897 (95% CI: 0.823–0.972) for the Wild-type strain, 0.668 (95% CI: 0.403–0.933) for the Delta strain, and 0.900 (95% CI: 0.828–0.972) for the Omicron strain for lymphocyte at discharge (Fig. 3J).

### 3.5. Differences of the levels of leukocytes and lymphocytes above and below the cutoff value in COVID-19 patients with wild-type, Delta and Omicron strains

A univariable COX model showed that HRs for mortality risk, with admission-stage leukocytes and lymphocytes levels above and below the cutoff value in COVID-19 patients with Wild-type, Delta, and Omicron strains, were 0.736 (95% CI: 0.402–1.349, *P* = .238) and 3.107 (95% CI: 1.707–5.656, *P* = .0002), 0.403 (95% CI: 0.071–2.306, *P* = .3073) and 0.605 (95% CI: 0.094–3.875, *P* = .5956), 0.386 (95% CI: 0.198–0.753, *P* = .0052) and 4.858 (95% CI: 2.491–9.473, *P* < .0001), respectively. Similarly, HRs for mortality risk, with discharge-stage leukocytes and lymphocytes levels above and below the cutoff value in COVID-19 patients with Wild-type, Delta and Omicron strains, were 0.009 (95% CI: 0.004–0.022, *P* < .0001) and 99.12 (95% CI: 44.53–220.6, *P* < .0001), 0.242 (95% CI: 0.043–1.360, *P* = .1072) and 0.313 (95% CI: 0.069–1.421, *P* = .1322), 0.051 (95% CI: 0.023–0.110, *P* < .0001) and 18.59 (95% CI: 8.916–38.78, *P* < .0001), respectively (Table 3).

**Table 3:** Differences of the levels of leukocytes and lymphocytes above and below the cutoff value in COVID-19 patients with wild-type, Delta, and Omicron strains.

		Admission levels		Discharge levels	
		HR (95% CI)	*P* value	HR (95% CI)	*P* value
Wild-type	*Leukocyte*				
	≤cutoff	Ref		Ref	
	>cutoff	0.736 (0.402–1.349)	.238	0.009 (0.004–0.022)	<.0001
	*Lymphocyte*				
	≤cutoff	Ref		Ref	
	>cutoff	3.107 (1.707–5.656)	.0002	99.12 (44.53–220.6)	<.0001
Delta	*Leukocyte*				
	≤cutoff	Ref		Ref	
	>cutoff	0.403 (0.071–2.306)	.3073	0.242 (0.043–1.360)	.1072
	*Lymphocyte*				
	≤cutoff	Ref		Ref	
	>cut-off	0.605 (0.094–3.875)	.5956	0.313 (0.069–1.421)	.1322
Omicron	*Leukocyte*				
	≤cut-off	Ref		Ref	
	>cut-off	0.386 (0.198–0.753)	.0052	0.051 (0.023–0.110)	<.0001
	*Lymphocyte*				
	≤cut-off	Ref		Ref	
	>cut-off	4.858 (2.491–9.473)	<.0001	18.59 (8.916–38.78)	<.0001

The variables were categorized into 2 groups according to the cut-off of each biomarker. The biomarkers were included as dichotomous variables in the univariable COX regression analysis.CI = confidence interval, HR = hazard ratio.

### 3.6. The cumulative survival rates of patient groups divided by cutoff, lower limit of normal (LLN), and upper limits of normal (ULN)

Kaplan–Meier curves showing the cumulative survival rates of patient groups divided by cutoff, LLN, and ULN. Optimal cutoff values were calculated based on ROC curves and improved prediction accuracy when compared with LLN and ULN values.

Kaplan–Meier curves showed that the admission-stage leukocytes levels above ULN values, in COVID-19 patients with Wild-type and Omicron strains, had a significantly higher risk of in-hospital mortality when compared with patients with below ULN value levels (Fig. 4A and C; *P* < .05). However, the discharge-stage leukocytes levels above newly established cutoff values, in COVID-19 patients with Wild-type and Omicron strains, had a significantly higher risk of in-hospital mortality when compared with patients with below cutoff value levels (Fig. 4D and F; *P* < .001). Unfortunately, the admission- and discharge-stage leukocytes levels, in COVID-19 patients with Delta strains, had not these characteristics (Fig. 4B and E).

**Figure 5.**
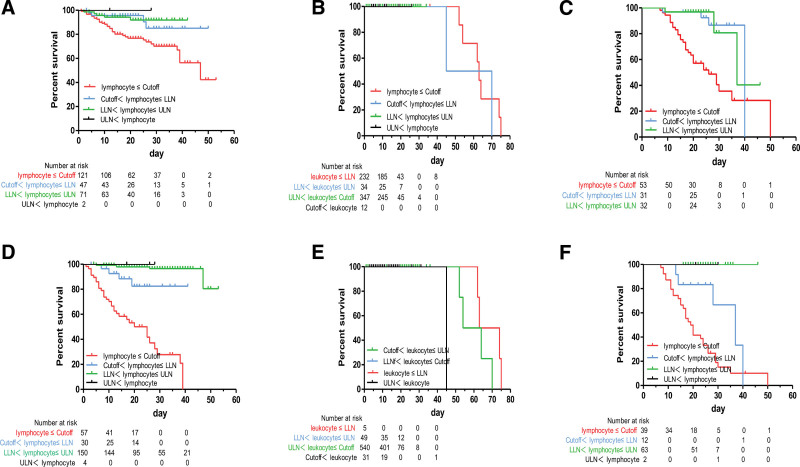
Kaplan–Meier curves showing the cumulative survival rates of patient groups divided by cutoff, LLN, and ULN. (A) Kaplan–Meier curve analysis based on the admission levels of lymphocyte in patients with Wild-type strain. (B) Kaplan–Meier curve analysis based on the admission levels of lymphocyte in patients with Delta strain. (C) Kaplan–Meier curve analysis based on the admission levels of lymphocyte in patients with Delta strain. (D) Kaplan–Meier curve analysis based on the discharge levels of lymphocyte in patients with Wild-type strain. (E) Kaplan–Meier curve analysis based on the discharge levels of lymphocyte in patients with Delta strain. (F) Kaplan–Meier curve analysis based on the discharge levels of lymphocyte in patients with Omicron strain. LLN = low limit of normal; ULN = upper limit of normal.

Kaplan–Meier curves also showed that the admission- and discharge-stage lymphocyte levels below newly established cutoff values, in COVID-19 patients with Wild-type and Omicron strains, had a significantly higher risk of in-hospital mortality when compared with patients with above cutoff value levels (Fig. 5A, B, E, and F; *P* < .01). Unfortunately, the admission- and discharge-stage lymphocytes levels, in COVID-19 patients with Delta strains, had not these characteristics (Fig. 5C and D).

## 4. Discussion

In the current study, we conducted a retrospective cross-sectional study to evaluate the changing trends, level differences, and prognostic performance of leukocyte and lymphocyte of different strains at admission and discharge may already exist in patients with COVID-19 infected with the Wild type, Delta and Omicron strains.

In the present study, the changes in the levels of leukocytes and lymphocytes exhibit a completely opposite trend in patients with COVID-19 infected with Wild type, Delta, and Omicron strains. The leukocyte level of survivors at discharge was the same or lower than that at admission, while the leukocyte level of non-survivors at discharge was significantly higher than that at admission; the lymphocyte level of survivors at discharge was significantly higher than that at admission, while the lymphocyte level of non-survivors at discharge was always at a low level. This finding was consistent with previous research. Wang et al^[16]^ discovered that an elevated white blood cell count was commonly observed in patients with SARS-CoV-2 infections, while most patients had marked lymphopenia, and non-survivors developed more severe lymphopenia over time. Sonaglioni et al^[17]^ investigated the clinical data of 74 patients with Omicron infection, and found that the neutrophil-to-lymphocyte ratio was the main prognostic indicators of in-hospital mortality. This also shows that leukocytes and lymphocytes were closely related to the prognosis of patients with COVID-19.

In the present study, the differences of leukocyte and lymphocyte of different strains at admission and discharge may already exist in patients with COVID-19 infected with Wild type, Delta, and Omicron strains. Among the survivors, the leukocyte of patients with COVID-19 infected with Delta at admission and discharge are lower than those of patients with COVID-19 infected with Wild type and Omicron strains; however, there was no difference in their levels among the non-survivors. Additionally, the lymphocyte of patients with COVID-19 infected with Delta at admission and discharge are higher than those of patients with COVID-19 infected with Wild type and Omicron strains among the survivors and non- survivors. There was inconsistent with previous research. Wang et al^[16]^ found that the lymphocyte count levels in the Omicron strain group were significantly higher than those in the Wild type and Delta strain group within 48 hours after admission; however, there was no difference in their leukocyte count levels among them. This phenomenon may be related to different populations and timing factors studied. The fact that during SARS-CoV-2 infection, to avoid being recognized and cleared by the human immune system, the anti-inflammatory response is increased, leukocytes and lymphocytes are negatively regulated, thus resulting in a decrease in leukocyte and lymphocyte counts.^[18–20]^ However, the specific roles of different strains of virus are still unknown in them.

In the present study, the performance of leukocyte and lymphocyte of different strains at admission and discharge in patients with COVID-19 infected with Wild type, Delta and Omicron strains was analyzed by Standard ROC curve. Overall performance of the leukocyte and lymphocyte were in Tables 1 and 2 and Figures 3 and 4. Compared to levels of the leukocyte and lymphocyte at admission (AUC_max_ ROC 62.8–86.1%), they presented better performance at discharge (AUC_max_ ROC 82.8–97.2%). Further analysis revealed that the leukocyte level of different strains at admission and discharge in patients with COVID-19 infected with Wild type, Delta, and Omicron strains presented similar performance. However, the lymphocyte level at admission and discharge in patients with COVID-19 infected with Omicron strains (AUC ROC 72.8–90.2%, 82.8–97.2%) presented better performance compared patients with COVID-19 infected with Wild type strains (AUC ROC 60.9–80.7%, 82.3–97.2%) and Delta strains (AUC ROC 56.1–84.7%, 40.3–93.3%). Moreover, the lymphocyte level at admission in patients with COVID-19 infected with Omicron strains shows an AUC = 0.815 that is near to some of the previously presented AUC to predict severity^[21]^ showing promising utility as a clinical tool for predicting disease severity.^[22,23]^

Then optimal cutoff value of leukocyte and lymphocyte of different strains at admission and discharge in patients with COVID-19 infected with Wild type, Delta, and Omicron strains were calculated based on ROC curves, and theirs the cutoff values were in Table 2, and to evaluate risk of in-hospital mortality. Univariate regression analyses further showed that differences of the levels of lymphocytes above and below the cutoff value for Wild-type and Omicron strains were statistically significant (*P* < .001), while there was not such difference in the cutoff value for Delta strains (*P* > .05). Kaplan–Meier curves showed that the leukocyte levels above newly established cutoff values and the lymphocyte levels below newly established cutoff values had a significantly higher risk of in-hospital mortality in COVID-19 patients with Wild-type and Omicron strains (*P* < .01), while there was not these characteristics in COVID-19 patients with Delta strains (*P* > .05). This indicates that the differences in leukocytes and lymphocytes from different strains may be due to the different risk of death in these patients. There was little relevant research. Previous research has found that the leukocyte and lymphocyte count in the Omicron strain group were higher than those in the Wild type and Delta strain groups and the differences were significant but still within the normal range.^[24]^ Like our research, the deeper significance of this difference was still unclear and further exploration was needed.

There are several limitations to this study. First, considering that the cohort infected with the Delta was included from a local epidemic, which was quickly controlled within about 1 month, the sample size was still relatively small, there were few patients who need to supplemental oxygen or Intensive Care Unit admission, and there were also fewer deaths. Smaller but statistically significant differences could have been missed due to the small sample size. Second, as this was a retrospective study, the lack of a randomized control group, so we cannot draw definitive conclusions. Thirdly, the absence of a negative control group for COVID-19 was a major defect in our research design. However, comparing leukocyte and lymphocyte level of different strains in patients with COVID-19 infected with Wild type, Delta, and Omicron strains would be valuable in driving the prevention and cure of COVID-19. Third, because the Wild-type, the Delta, and the Omicron strain were included in the 2 hospitals, and the interval is more than 2 years, initial blood samples were also not processed in a centralized same laboratory; therefore, there are some differences in inspection methods and results. However, the clinical testing protocol in China is conducted in a standardized manner, and any possible intraassay variability would have been distributed equally across the entire cohort. Despite this, the present results are in line with the previously study of different VOCs in COVID-19.^[12,25]^ Therefore, we believe that the study here is meaningful. In addition, more detailed patient information, larger sample size, particularly regarding clinical data of severe and critical, was very necessary at the time of analysis. Greater efforts should be made to solve these problems in the future research.

## 5. Conclusions

The levels of leukocyte and lymphocyte at admission and discharge in patients with COVID-19 infected with the Wild type, Delta, and Omicron strains may be differences among strains, which indicates different death risks. Our research may help clinicians identify patients with a poor prognosis and guide treatment strategies for SARS-CoV-2 infection.

## Acknowledgements

We owed our earnest thanks to all the staffs of Xi’an Chest Hospital and Wuhan Huoshenshan Hospital who remained at their posts during the COVID-19 epidemic.

## Author contributions

**Data curation:** Yanjun Zhao, Xing Gu.

**Formal analysis:** Wenjie Li, Yaqin Chai.

**Funding acquisition:** Hongjun Zhang.

**Software:** Hongjun Zhang.

**Writing – original draft:** Hongjun Zhang.

**Writing – review & editing:** Xing Gu.
